# Cardiac endothelial sarcoma with hypereosinophilia of children: a case report

**DOI:** 10.1186/s13019-023-02206-4

**Published:** 2023-04-07

**Authors:** Wei Zhang, Mengyuan He, Qiang Wang

**Affiliations:** 1 Department of Cardiac Surgery, Tianjin Chest Hospital, Tianjin, China; 2 Tianjin Medical University, Tianjin, China

**Keywords:** Hypereosinophilia, Eosinophilia, Cardiac tumor, Malignancy, Children

## Abstract

Cardiac malignancies in children are extremely rare and they with hypereosinophilia are rather relatively uncommon. The majority of individuals may survive over the long term even with heart tumors provided they don't have any significant symptoms and their hemodynamics are unaffected. But we should nevertheless be aware of them, especially when they are coupled with persistent hypereosinophilia and the development of a hemodynamic anomaly. The case of a malignant heart tumor with hypereosinophilia in a 13-year-old girl is presented in this paper. She exhibited an echocardiographic deficit and a heart murmur. Additionally, it was difficult to treat her hypereosinophilia. Nevertheless, it was resolved the day after the operation. We presume that there is a certain relationship between them. This study gives clinicians a wide range of options for analyzing the connections between malignancy and hypereosinophilia.

## Case report

A 13-year-old girl was admitted to our hospital with intermittent coughing for a month that had no apparent explanation and no other symptoms.

**Fig. 1:**
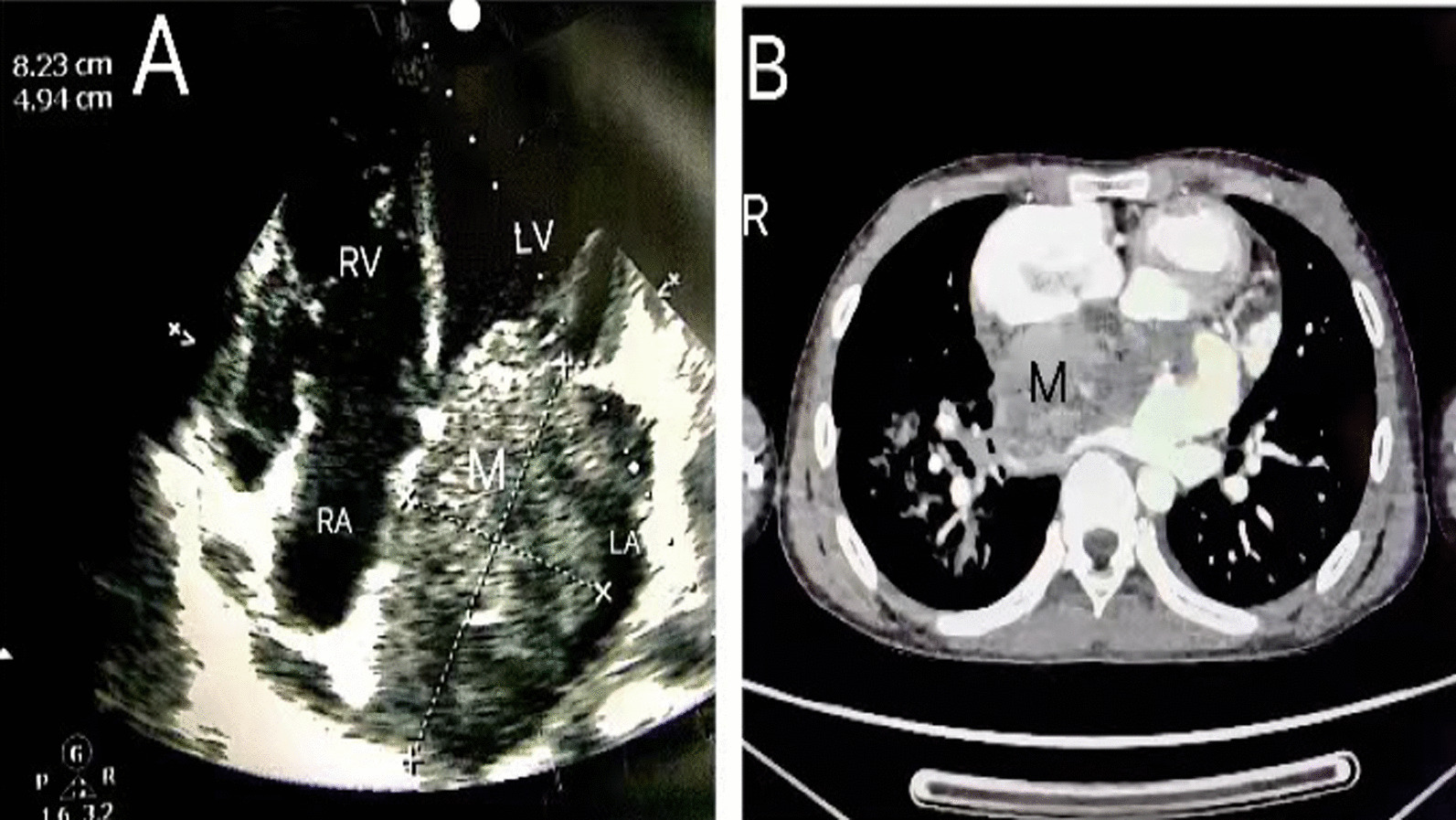
Transthoracic echocardiogram (the apical four chamber view) and Cardiac computed tomography. **A** Transthoracic echocardiogram. **B** Cardiac computed tomography. *LA* Left atrium, *LV* Left ventricle, *M* Mass. *RA* Right atrium, *RV* Right ventricle

She was diagnosed with pneumonia, four days earlier, at the Tianjin Children's Hospital. After being admitted, the patient received anti-infection, and anti-virus treatments. The white blood cell (WBC) count from the peripheral blood analysis was 43,900/mm^3^ (reference range: 4600–11,300/mm^3^), with 24 percent of those cells being eosinophilic granulocytes (absolute eosinophil count: 10,450/mm^3^). Her eosinophils did not resolve the following therapy with allopurinol and methylprednisolone [1]. Additionally, a 2/6 -3/6 murmur could be heard in the precardiac region, thus echocardiography was performed: “left atrial myxoma?”. To better clarify the nature of the lesion, an enhanced CT scan (Fig. 1) was performed. A larger soft-tissue mass was seen in the LA that had a substantial amount of heterogeneous enhancement on the enhancement scan. It protruded into the mitral valve region, was locally expanded into the right pulmonary vein trunk, and was only partially discernible from the mitral valve. Her parents rejected cardiac MRI due to financial problems.

The leukocyte count was 45,540/mm^3^ with an absolute eosinophil count of 12,590/mm^3^(27.6% eosinophils). Tachycardia was seen on the electrocardiogram (ECG), and RV5 = RV6 and some leads had a notched P wave. And An 8.2 × 4.9 cm space-occupying lesion in the LA, which occupied around 80% of the LA, was detected by echocardiography (Fig. 1). Its base was broad, linked to all the interatrial septum, and attached to the anterior mitral valve leaflet. A tiny fraction of it was extremely mobile and obstructed the mitral orifice with respiratory and the majority were comparatively less mobile. Her left ventricle ejection fraction was 68% and the lesion has caused moderate to severe mitral and tricuspid regurgitation.

**Fig. 2:**
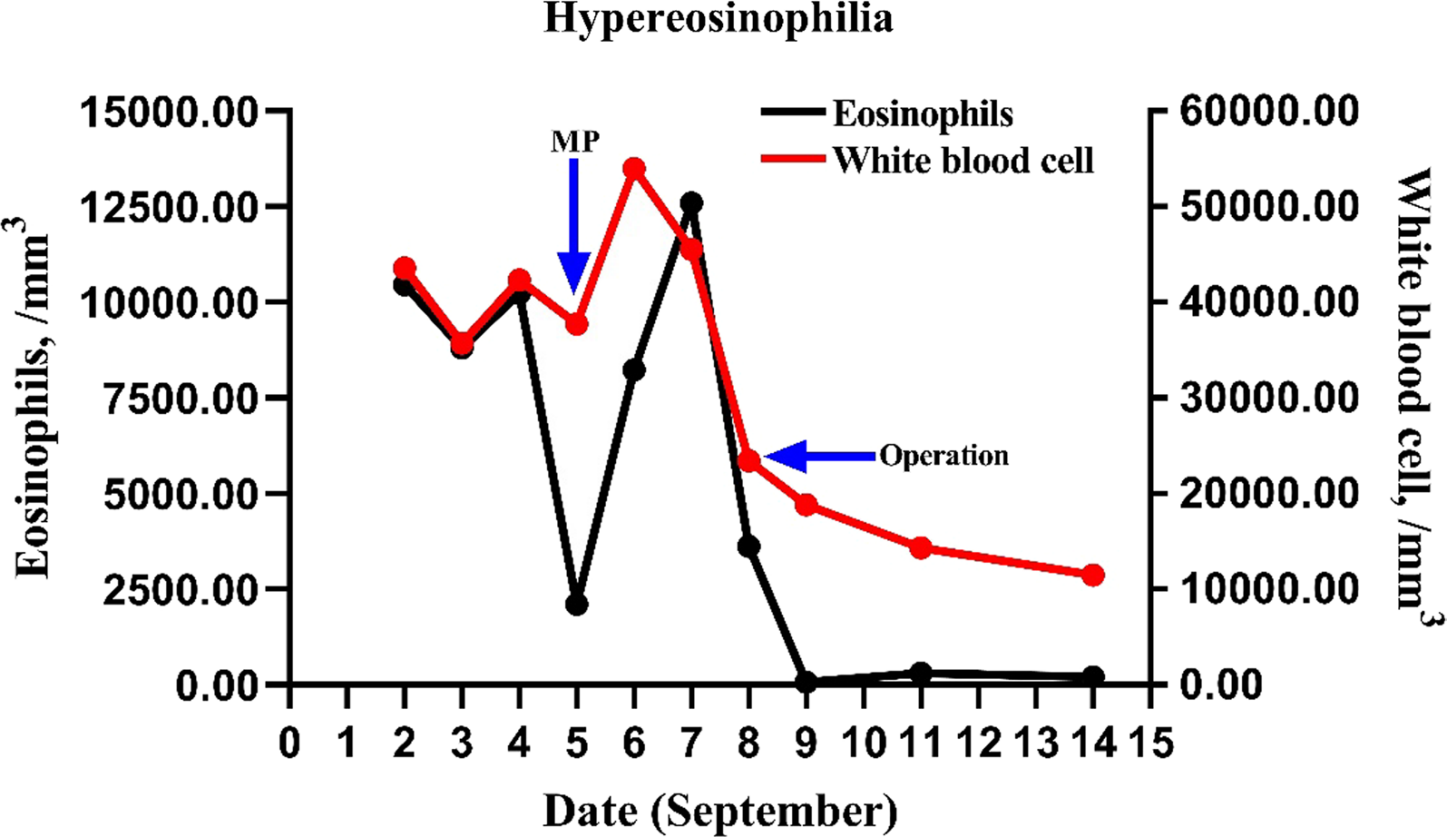
White blood cell and eosinophil counts. MP, treatment with methylprednisolone

**Fig. 3:**
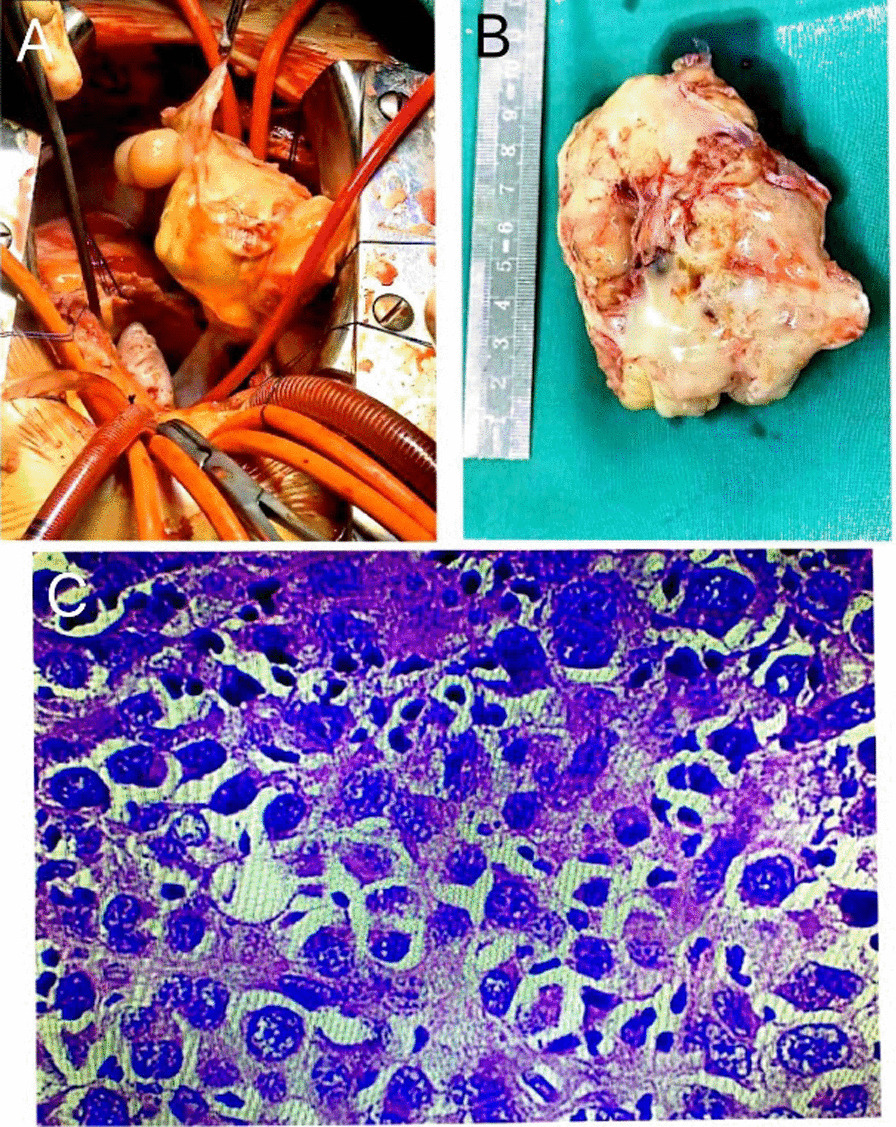
Gross view and Pathological findings. **A** and **B** Macroscopy of the tumor. **C** Photomicrograph of a hematoxylin–eosin-stained section of the atrial mass, original magnifications 10 × and 40 × , respectively

She was sent to the surgery room to have the intracardiac tumor excised and the mitral valve replaced while under general anesthesia since the development of a hemodynamic anomaly. The patient subsequently had a profound decrement in the eosinophil count, the absolute eosinophil count was 70/mm^3^ (0.4% eosinophils) and the WBC count was 18,820/mm^3^ (Fig. 2). The pathological diagnosis of the atrial mass showed that it was a malignant small round cell tumor (8.5*6*4.5 cm in size) and the tumor cells were positive for vimentin, P53, and PLAP, partially positive for desmin, CD31, CD68, S-100, LCA, and negative for CK(P), sarcomeric actin, CD34, HMB45 (Fig. 3), which finally was diagnosed as endothelial sarcoma.

She was discharged from the hospital in good clinical condition and advised to seek additional therapy at an oncology institution, but she and her parents declined. The patient eventually died from brain herniation, and cerebral bleeding 3 months later, which might result from resulted from metastases. The requirement to examine the deceased person's body was denied.

## Discussion

Hypereosinophilia is defined as a peripheral blood eosinophil count exceeding 1500/mm^3^ [1]. Tumors with it are usually brought on by mucin-secreting epithelium, such as uterine, bronchial, pancreatic, or intestinal tumors [2]. Primary cardiac tumors in infants and children are extremely rare and the majority of pediatric cardiac tumors are primary benign cardiac tumors, with myxoma, fibroma, and rhabdomyoma being the most prevalent varieties, while malignant, especially endothelial sarcoma, is rare [3–5].

Typically, the location, size, quantity, invasiveness, and pace of growth of tumors are correlated with symptoms. Additionally, a study found that the majority of individuals may survive over the long term even with heart tumors provided they don't have any significant symptoms and their hemodynamics are unaffected [3]. And a multicenter study suggests that surgery is advocated if symptoms, ECG abnormalities, echocardiographic impairment, or ECG abnormalities are present [6]. The patient in our research exhibited an echocardiographic deficit, an irregular ECG, and a heart murmur. Additionally, it was difficult to treat her hypereosinophilia. Nevertheless, it was resolved the next day after the operation. We thus presume that there is a certain relationship between cardiac endothelial sarcoma and hypereosinophilia.

There are several different causes of hypereosinophilia, which are often categorized into four broad groups: familial, secondary (reactive), primary, and idiopathic [7–9]. It has occasionally been observed in individuals with a range of solid malignant tumors, which is one of the secondary causes. Heart tumors are less prevalent than lung and stomach malignancies in previously documented cases of hypereosinophilia in solid tumors [10]. Among the reported cardiac tumors, the vast majority are benign cardiac tumors [11, 12]. And only one prior case of hypereosinophilia associated with malignant cardiac tumors has been described in the past 20 years [13]. A 20-year-old male with fever, acute dysarthria, and right-sided hemiplegia was the patient, in that case, it was revealed. His WBC count was 34,000/mm^3^ with 8% eosinophils (absolute eosinophil count: 2,720/mm^3^). The pathologic diagnosis was rhabdomyosarcoma. Four weeks following the surgery, he had a cutaneous metastasis to the right temple and metastatic involvement of the retroperitoneum.

Wei et al. reported two patients, who were diagnosed with a renal tumor, had persistent eosinophilia of pre-operation and normal eosinophilic granulocytes following surgery [14]. And a case of a 72-year-old man demonstrated that non-small-cell lung cancer was associated with excessive eosinophilia and resolution of it after surgical resection [15]. Additionally, the solid intestinal tumors had been observed by Caruso et al. presented eosinophilia in the blood count and it turned to normal post-operation [16]. Similarly, this study showed that it was a remarkable return to normal eosinophil numbers after surgery.

There are a variety of mechanisms of eosinophils to promote the development of tumors [17]: (1)Immunosuppressive regulatory T cells may move more easily to the tumor microenvironment when they are stimulated by the CC-chemokine ligand 22 produced by eosinophils; (2)Indoleamine 2,3-dioxygenase expressed by them, catalyzes the oxidation degradation of l-tryptophan, which leads to suppressing effector T cell responses while inducing suppressive immunity; (3) A large number of growth factors produced and secreted by them, such as epidermal growth factor which can promote tumor cell growth, and transforming growth factor-β1 which induces the epithelial-mesenchymal transition; (4) By secreting matrix metalloproteinases like MMP2 and MMP9, eosinophils cause the matrix to restructure, which may also make it easier for the seeding of metastatic lesions.

In conclusion, Cardiac malignancy in children is extremely rare, and the symptoms may not be immediately apparent, but we should nevertheless be aware of them, especially when they are coupled with persistent eosinophilia and echocardiogram indicates space-occupying lesions. Additionally, this study gives clinicians a wide range of options for analyzing the connections between malignancy and hypereosinophilia.

## Data Availability

Data sharing is not applicable to this article as no datasets were generated or analysed during the current study.
